# IL-35 Is a Novel Responsive Anti-inflammatory Cytokine — A New System of Categorizing Anti-inflammatory Cytokines

**DOI:** 10.1371/journal.pone.0033628

**Published:** 2012-03-16

**Authors:** Xinyuan Li, Jietang Mai, Anthony Virtue, Ying Yin, Ren Gong, Xiaojin Sha, Stefanie Gutchigian, Andrew Frisch, Imani Hodge, Xiaohua Jiang, Hong Wang, Xiao-Feng Yang

**Affiliations:** Department of Pharmacology and Cardiovascular Research Center, Temple University School of Medicine, Philadelphia, Pennsylvania, United States of America; French National Centre for Scientific Research, France

## Abstract

It remains unknown whether newly identified anti-inflammatory/immunosuppressive cytokine interleukin-35 (IL-35) is different from other anti-inflammatory cytokines such as IL-10 and transforming growth factor (TGF)-β in terms of inhibition of inflammation initiation and suppression of full-blown inflammation. Using experimental database mining and statistical analysis methods we developed, we examined the tissue expression profiles and regulatory mechanisms of IL-35 in comparison to other anti-inflammatory cytokines. Our results suggest that in contrast to TGF-β, IL-35 is not constitutively expressed in human tissues but it is inducible in response to inflammatory stimuli. We also provide structural evidence that AU-rich element (ARE) binding proteins and microRNAs target IL-35 subunit transcripts, by which IL-35 may achieve non-constitutive expression status. Furthermore, we propose a new system to categorize anti-inflammatory cytokines into two groups: (1) the house-keeping cytokines, such as TGF-β, inhibit the initiation of inflammation whereas (2) the responsive cytokines including IL-35 suppress inflammation in full-blown stage. Our in-depth analyses of molecular events that regulate the production of IL-35 as well as the new categorization system of anti-inflammatory cytokines are important for the design of new strategies of immune therapies.

## Introduction

In the past ten years, significant progress has been made in characterizing the roles of CD4^+^CD25^high^Foxp3^+^ regulatory T cells (Tregs) in inhibition of various types of inflammation and immunological diseases [1], [2], [3], [4]. However, it remains poorly defined what anti-inflammatory/immunosuppressive mechanisms healthy individuals have in order to maintain normal tissue functions such as by preventing the initiation of inflammatory process and inhibiting the progression of inflammation.

CD4^+^ T helper cells (Th) play essential roles in regulating inflammation and immune responses via differentiation into various Th functional subtypes, including Th1, Th2, Th17, Th9, Th22, follicular Th, and Tregs [5]. The majority of Th cell functions are fulfilled via the secretion of various cytokines, which can play a dual role in regulating chronic inflammation and autoimmune diseases [6]. Proinflammatory and Th1-related cytokines such as IL-1 and IL-18 promote the development and progression of inflammation and immune responses [7], [8]. However, anti-inflammatory and Tregs-related cytokines such as IL-10 and TGF-β exert clear anti-inflammatory activities [9]. It therefore stands to reason that patients with autoimmune diseases [10], angina [11], or familial hypercholesterolemia [12] have lower serum IL-10 levels than healthy controls. In fact, the transfer of human IL-10 or intracerebral injection of IL-10 significantly inhibit experimental autoimmune encephalomyelitis [13], [14]. These findings suggest that immunosuppressive/anti-inflammatory cytokines play a critical role in the inhibition of inflammation and autoimmune diseases.

Interestingly, IL-35 has been identified as a novel immunosuppressive/anti-inflammatory cytokine. It is a dimeric protein with two subunits, IL-12A and Epstein-Barr virus induced 3 (EBI3) [15], [16]. Secretion of IL-35 has only been confirmed in non-stimulated mouse Tregs [16] and in stimulated human Tregs [17] but not detected in non-stimulated human Tregs [18]. In addition to T cells, other cells in vessels, such as endothelial cells and vascular smooth muscle cells, also generate various inflammation-regulating cytokines [19]. However, the question of whether non-T cells such as vascular cells express IL-35 remains.

Thus, despite significant progress, several important knowledge gaps exist: First, how IL-35 is expressed in human and mouse tissues; Second, if IL-35 is not constitutively expressed, then what are the transcriptional and post-transcriptional mechanisms controlling its induction and degradation; and Third, whether IL-35 is temporally different from TGF-β and IL-10 in the inhibition of inflammation in non-inflammatory or inflammatory stages. In this study, we hypothesized that anti-inflammatory/immunosuppressive cytokines including IL-35 have differential expression in various tissues under non-stimulated conditions. Using database mining and statistical analysis methods that we developed [20], [21], we examined the expression of anti-inflammatory cytokines in numerous tissues from a panoramic viewpoint. Furthermore, we examined the potential molecular mechanisms regulating IL-35 expression. The in-depth analysis of the expression patterns of IL-35 in comparison to that of other anti-inflammatory cytokines could provide novel avenues for innovative therapeutic treatments for inflammation and autoimmune diseases.

## Methods

### Tissue expression profiles of genes encoding anti-inflammatory cytokines and their receptors

**Figure 1:**
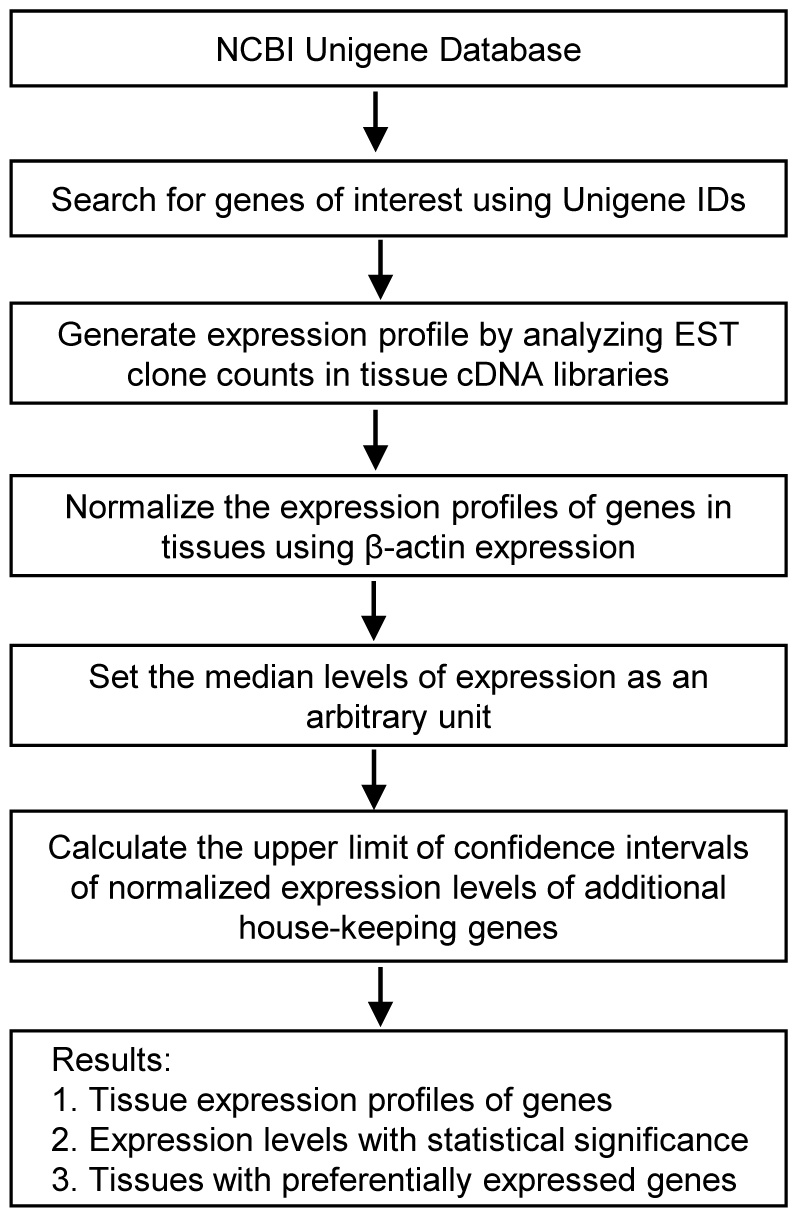
Flow chart of database mining strategy that was used to generate tissue expression profiles of genes. NCBI: National Center of Biotechnology Information; IDs: Identifications; EST: Expressed sequence tag.

**Figure 2:**
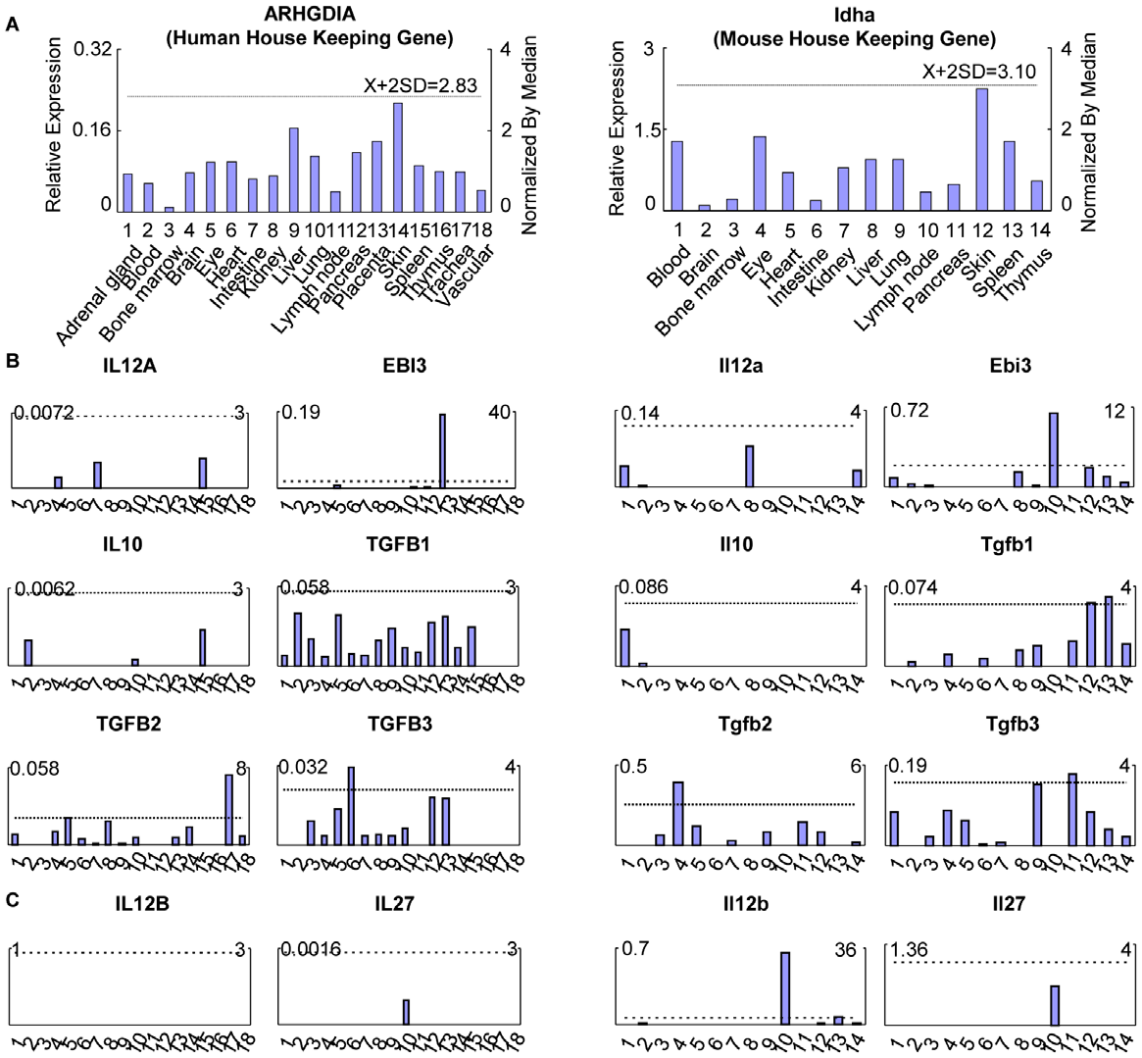
The gene expression profiles found in human and mouse tissues. A. Data presentation format (The data presented in X-, Y-axis, and tissue order of ARHGDIA and Idha are applied to all the human and mouse genes examined respectively). As an example, the gene expression profiles of human housekeeping gene Rho GDP dissociation inhibitor (GDI) alpha (ARHGDIA) in the eighteen tissues are presented, with the tissue names and position numbers shown on the X-axis. The gene expression data were normalized by the β-actin (Hs. 520640) expression data from the same tissue, which are presented on the left Y-axis. The expression ratios among tissues were generated by normalizing the arbitrary units of the gene in the tissues with the median level of the arbitrary units of the gene in all the tissues which are presented on the right Y-axis. In order to define confidence intervals for statistically higher expression levels of given genes, we calculated the confidence intervals of tissue expression for three housekeeping genes [the mean X+2×standard deviations (SD) = 2.83] including ARHGDIA (Hs. 159161), glyceraldehyde-3-phosphate dehydrogenase (GAPDH, Hs. 544577), and ribosomal protein S27a (RPS27A, Hs. 311640). The expression variations of given genes in tissues, when they were larger than 2.83-fold, were defined as the high expression levels with statistical significance (the right Y-axis). To define confidence intervals for statistically higher expression levels of given genes in 14 mouse tissues, we calculated the confidence intervals of tissue expression [the mean X+2×standard deviations (SD) = 3.1] for three mouse house keeping genes including Lactate dehydrogenase A (Ldha, Mm. 29324), non-POU-domain-containing octamer binding protein (Nono, Mm. 280069), and ribosomal protein L32 (Rpl32, Mm. 104368). The expression variations of given genes in tissues, when they were larger than 3.1-fold, were defined as the high expression levels with statistical significance (the right Y-axis). B. The expression profiles of suppressive cytokines in human (Left 2 columns, with cytokine members designated with capital letters) and mouse (right 2 columns, with cytokine members designated with lowercase letters) tissues. C. The expression profiles of IL-35 related subunits in human and mouse tissue.

An experimental data mining strategy (Fig. 1), as we reported [20], [21], was used to analyze the expression profiles of mRNA transcripts of genes in cardiovascular and other tissues in humans and mice by mining experimentally verified human and mouse mRNA expressions in the expressed sequence tag (EST) databases of the National Institutes of Health (NIH)/National Center of Biotechnology Information (NCBI) UniGene (http://www.ncbi.nlm.nih.gov/sites/entrez?db=unigene). Transcripts per million of genes of interest were normalized with that of house-keeping β-actin in each given tissue to calculate the arbitrary units of gene expression. A confidence interval of the expression variation of house-keeping genes was generated by calculating the mean plus two times that of the standard deviation of the arbitrary units of three randomly selected house-keeping genes (PRS27A, GADPH, and ARHGDIA in human; Ldha, Nono, and Rpl32 in mouse) normalized by β-actin in the given tissues (Fig. 2A). If the expression variation of a given gene in the tissues was larger than the upper limit of the confidence interval (the mean plus two times the standard deviation) of the house-keeping genes, the high expression levels of genes in the tissues were considered statistically significant. Gene transcripts lower than one per million were technically presented as no expression.

### Analysis of transcription factor binding sites in the promoters of anti-inflammatory cytokines

**Figure 3:**
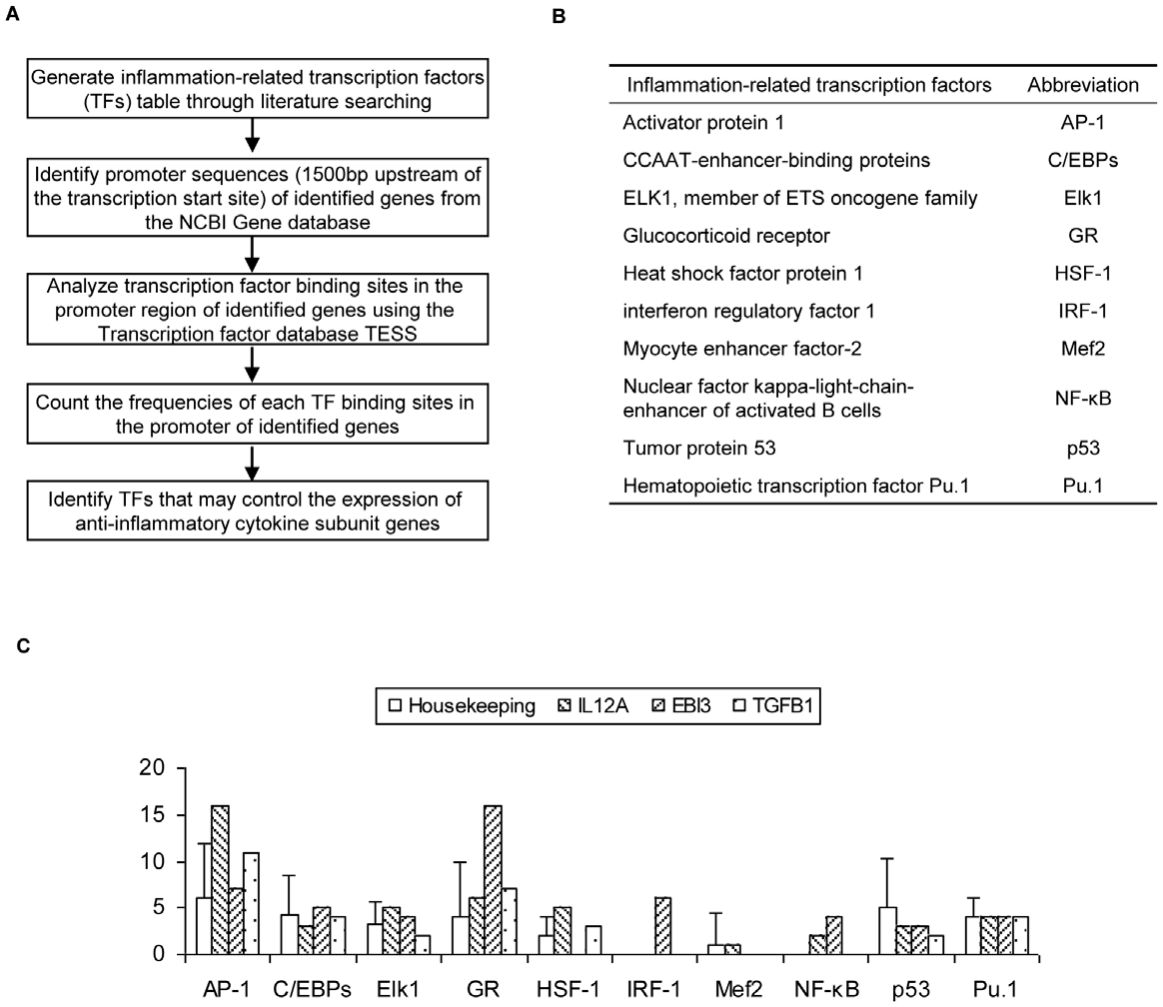
Transcription factor binding frequencies in the promoter region of IL-35. The promoter sequences (1500 base pair upstream of the transcription start site) of 3 housekeeping genes (ACTB, GAPDH, ARHGDIA), IL12A, EBI3, and TGFB1 were retrieved from the NIH/NCBI Entrez Gene database, and were analyzed using TESS to determine the frequencies of 10 Transcription factors (TFs). The binding frequencies of the 10 TFs in the promoter region of each gene were counted. Confidence interval was set by using the mean+2×standard deviation (SD) of the TF binding frequencies in the promoter of 3 housekeeping genes. The binding frequency which is higher than the uppermost confidence interval (p<0.05) is considered significant.

The promoter regions of targeted genes, defined as 1,500 bases upstream of the transcription start site, were retrieved from the NIH/NCBI Entrez Gene database (http://www.ncbi.nlm.nih.gov/gene). In addition, the promoter region was analyzed with the widely-used transcription factor database TESS (http://www.cbil.upenn.edu/cgi-bin/tess/tess) to analyze the frequencies of 10 inflammation related transcription factor binding sites (Fig. 3A).

### Alternative spliced isoforms of anti-inflammatory cytokines

The presence and features of alternative promoters and alternatively spliced isoforms of each gene were examined with the AceView database of NIH/NCBI (http://www.ncbi.nlm.nih.gov/IEB/Research/Acembly/index.html).

### Correlation of the ratios of tissue SAH versus SAM concentrations with the expression levels of anti-inflammatory cytokines and receptors

The concentrations of S-adenosylmethionine (SAM) levels over S-adenosylhomocysteine (SAH) levels were determined previously by Ueland's group [22] in tissues from adult male mice. Tissue SAM/SAH ratios were used for further comparison and regression analyses. Simple linear regression analyses were performed by plotting mRNA levels of individual genes against the SAM/SAH ratios in seven mouse tissues including the brain, kidney, liver, spleen, heart, lung, and thymus. Multivariable regression analyses were then performed to evaluate the effect of SAM/SAH ratios on the expressions of anti-inflammatory cytokines and receptors [20].

### Presence of adenine and uracil nucleotide-rich (AU-rich) elements in 3′-untranslated regions of anti-inflammatory cytokines

The genes of interest were searched in the UTRdb (http://utrdb.ba.itb.cnr.it/search) at the Institute for Biomedical Technologies, University of Bari for the existence of functional motifs and signals in the 3′-untranslated regions (3′UTR) of each mRNA. The presence of AU-rich elements in the 3′UTRs was searched using the AU-rich Element Containing mRNA Database (ARED 3.0) (http://brp.kfshrc.edu.sa/ARED).

### MicroRNA interactions with the mRNAs of anti-inflammatory cytokines

The potential interactions of mRNAs from the genes of interest with microRNAs were examined using the Bioinformatics and Research Computing software TargetScan (http://www.targetscan.org) from the Whitehead Institute for Biomedical Research in the Massachusetts Institute of Technology (MIT). The significance of MicroRNA binding to the genes of interest was determined using the confidence intervals generated from the microRNAs within Tarbase, an experimentally verified MicroRNA online database (http://diana.cslab.ece.ntua.gr/tarbase). Briefly, human microRNAs, which were single site effective and luciferase assay-confirmed, were used to ensure that the interactions of the microRNAs were specific to their respective mRNA targets. 27 microRNAs that met the criteria were selected and evaluated in TargetScan to construct the intervals and set the lower limit for the context values and score percentile as we reported [23]. MicroRNAs with the context score of 70% or higher and the context value of −0.22 or lower were determined to be significant.

## Results

### 1. IL-35 is not constitutively expressed in non-stimulated human tissues, whereas TGF-βs have high expression levels

We hypothesized that to keep inflammation in check, various tissues express certain levels of anti-inflammatory cytokines under physiological conditions. To examine this hypothesis, a database mining method we developed (Fig. 1) [21], was used to examine experimentally verified expression profiles of mRNA transcripts of anti-inflammatory cytokines (Table 1) and receptors (Table S1) in NCBI-UniGene database. The expression of three sets of genes in 18 human tissues and 14 mouse tissues were examined (fewer mouse tissues were examined due to the fact that gene expression data for four of the mouse tissue counterparts were not available in the database). The first set (Fig. 2B) had six anti-inflammatory cytokines/cytokine subunits [IL-35 (IL-12A, EBI3), IL-10, three isoforms of TGF-β (TGF-β1, TGF-β2, TGF-β3)]; the second set (Fig. 2C) included two IL-35-related subunits (IL-12B, IL-27); and the third set (Figure S1) included IL-35 receptor subunits (IL-12Rβ2, IL-6ST), IL-10 receptor (IL-10RA, IL-10RB) and TGF-β receptor (TGF-βR1, TGF-βR2). Of the 18 human tissues examined (Table 2), the expression of the first IL-35 subunit, IL-12A, was only found in brain, intestine, and spleen at very low levels, whereas the second IL-35 subunit, EBI3, was expressed at low levels in eye, lymph node, and pancreas, with high levels in the placenta. IL-10 was also expressed at very low levels only in blood, lung, and spleen but not in heart or vessels. In comparison, all three isoforms of TGF-β were highly expressed in most tissues including heart tissue. TGF-β1 was widely expressed in all tissues except thymus, trachea, and vasculature, suggesting anti-inflammatory and other physiological functions of these cytokines in human tissues. However, TGF-β2 was significantly expressed in the trachea but not expressed in immune tissues (bone marrow, lymph node, spleen, and thymus), and TGF-β3 was expressed highly in heart but not in lymph node, spleen, and thymus, suggesting that both TGF-β2 and TGF-β3 may be mainly functional in non-immune tissues. In addition, the expression patterns of anti-inflammatory cytokines in mouse tissues were similar to their human counterparts except for IL-35 (IL-12A and EBI3). IL-12A and EBI3 had significantly higher expression levels in mice when compared to humans (Fig. 2B). Furthermore, IL-12A was found in mouse blood, bone marrow, liver, and thymus, whereas EBI3 was expressed in mouse blood, bone marrow, liver, skin, spleen, thymus, and high levels in mouse lymph node. Taken together, anti-inflammatory cytokines IL-35 and IL-10 had very low expressions in non-stimulated tissues, whereas TGF-βs had high expression levels, suggesting that the latter cytokines, but not the former ones, are required for anti-inflammatory functions under non-stimulated “house-keeping” conditions. This conclusion is further supported by gene knockout studies in mice. The gene deficiencies of IL-12A, EBI3, and IL-10 were not lethal to mice in contrast to the massive inflammation leading to organ failure and death observed in TGF-β1 knockout mice (Table 3).

**Table 1:** The Unigene ID of human and mouse genes that were examined.

UniGene Name	Cytokine/Receptor Subunit	Related cytokine	Unigene ID/Human	Unigene ID/Mouse
**Anti-inflammatory Cytokine**				
IL12A	Interleukin 12 A	IL35	Hs. 673	Mm.103783
EBI3	Epstein-Barr induced virus gene 3	IL35	Hs. 501452	Mm. 256798
IL10	Interleukin 10	IL10	Hs. 193717	Mm. 874
TGFB1	Transforming growth factor, beta 1	TGFβ	Hs. 645227	Mm. 248380
TGFB2	Transforming growth factor, beta 2	TGFβ	Hs. 133379	Mm. 18213
TGFB3	Transforming growth factor, beta 3	TGFβ	Hs. 592317	Mm. 3992
**Other IL-35 related subunits**				
IL12B	Interleukin 12 B	IL12	Hs. 674	Mm. 239707
IL27	Interleukin 27	IL27	Hs. 582111	Mm. 222632

**Table 2:** Anti-inflammatory cytokines are differentially expressed in human and mouse tissues.

Tissues	Human Expression	Mouse Expression
Adrenal Gland	TGFB1, TGFB2	
Blood	IL10, TGFB1	Il12a, Ebi3, Il10, Tgfb3
Bone Marrow	TGFB1, TGFB3	Il12a, Ebi3, Il10, Tgfb1, Tgfb3
Brain	IL12A, TGFB1, TGFB2, TGFB3	Ebi3, Tgfb2, Tgfb3
Eye	EBI3, TGFB1, TGFB2, TGFB3	Tgfb1, Tgfb2, Tgfb3
Heart	TGFB1, TGFB2, TGFB3	Tgfb2, Tgfb3
Intestine	IL12A, TGFB1, TGFB2, TGFB3	Ebi3, Tgfb1, Tgfb3
Kidney	TGFB1, TGFB2, TGFB3	Tgfb2, Tgfb3
Liver	TGFB1, TGFB2, TGFB3	Il12a, Ebi3, Tgfb1
Lung	IL10, TGFB1, TGFB2, TGFB3	Ebi3, Tgfb1, Tgfb2, Tgfb3
Lymph Node	EBI3, TGFB1	Ebi3
Pancreas	EBI3, TGFB1, TGFB3	Tgfb1, Tgfb2, Tgfb3
Placenta	EBI3, TGFB1, TGFB2, TGFB3	
Skin	TGFB1, TGFB2	Ebi3, Tgfb1, Tgfb2, Tgfb3
Spleen	IL12A, IL10, TGFB1	Ebi3, Tgfb1, Tgfb3
Thymus	-	Il12a, Ebi3, Tgfb1, Tgfb2, Tgfb3
Trachea	TGFB2	
Vascular	TGFB2	

**Table 3:** Effects of anti-inflammatory cytokine gene knockout (−/−) in mice.

Gene−/−	Viable	Reported phenotype	PMID
IL12−/−	Yes	Display normal development	8766560
EBI3−/−	Yes	No overt autoimmunity or inflammatory disease	12482940
IL10−/−	Yes	Only a local inflammation limited to the proximal colon	8402911
TGFB1−/−	No	Multifocal inflammatory cell response and tissue necrosis	1436033
TGFB2−/−	No	Perinatal mortality	9217007
TGFB3−/−	No	Die within 20 hours of birth	7493022

IL-35 signals through heterodimeric receptor made of IL-12Rβ2 and gp130 (encoded by IL-6ST) or homodimers of each chain [24]. IL-6ST was ubiquitously expressed in all the tissues examined (Figure S1). Meanwhile IL-12Rβ2 was expressed in seven tissues but not in heart and vascular tissue. It is therefore conceivable to speculate that the use of gp130-gp130 homodimers may provide a mechanism by which IL-35 could affect tissues that do not express IL-12Rβ2.

Heart and vascular tissue expressed IL-10RA, IL-10RB, TGF-βR1, and TGF-βR2 in human (Figure S1). Similarly, mouse heart also expressed IL-10 and TGF-β receptor complexes. IL-10 acts through a transmembrane receptor complex, which is composed of IL-10RA and IL-10RB. It plays an important role in the control of both innate and adaptive immunity [25]. IL-10 deficiency promotes atherosclerotic lesion formation, characterized by increased infiltration of inflammatory cells and increased production of proinflammatory cytokines [26]. TGF-β isoforms including TGF-β1, TGF-β2, and TGF-β3 were expressed in the vessel wall. They signal through TGF-βR1 and TGF-βR2 via activating Smad-dependent and Smad-independent signals and play a key role in atherosclerosis [27]. These results suggest that cardiovascular tissues express IL-10 and TGF-β receptors and are able to accept these anti-inflammatory cytokine signals to inhibit inflammation.

### 2. IL-35 is a responsive anti-inflammatory cytokine that could be induced by proinflammatory cytokines in non-T cells

As a new member of the IL-12 heterodimeric cytokine family, IL-35 shares subunit IL-12A with IL-12 and subunit EBI3 with IL-27. We classified the tissues into two tiers based on their expression of the heterodimeric cytokine subunits (Table 4). Thus, tissues that expressed both subunits of a given heterodimeric cytokine were placed in the first tier of “ready to go” status. Tissues that did not express one or both the subunits were placed in the second tier of “inducible” status. For IL-35 expression, mouse blood, bone marrow, liver, and thymus tissues, but none of the human tissues, were in the first tier. Since secretion of IL-35 has only been confirmed in non-stimulated mouse Tregs [15] but not in non-stimulated human Tregs [18], our results support the idea that the expressions of IL-35 in tissues are different between human and mouse. It should be noted, that it is unknown whether IL-35 expression in these four mouse tissues is due to potential high levels of Tregs in these tissues. In addition, for the two other IL-35 related cytokines, IL-12 and IL-27, none of the human tissues were in the first tier. For mouse IL-27, only lymph node was in the first tier. While for mouse IL-12, bone marrow and thymus were in the first tier. Taken together, the results suggest that IL-35 is not constitutively expressed in human tissues but may be an inducible anti-inflammatory cytokine that may control full-blown inflammation.

**Table 4:** The two-tier expression status of IL-35 and related cytokines is identified.

Cytokine	Human tissue	Mouse tissue
**First tier (“ready to go” expression status with two cytokine subunits)**		
IL-35	-	Blood, Bone Marrow, Liver, Thymus
IL-12	-	Bone Marrow, Thymus
IL-27	-	Bone Marrow, Lymph Node
**Second tier (“inducible” expression status that requires up-regulation of at least one cytokine subunit)**		
IL-35	All the tissue	Brain, Eye, Heart, Intestine, Kidney, Lung, Lymph Node, Pancreas, Skin, Spleen
IL-12	All the tissue	Blood, Brain, Eye, Heart, Intestine, Kidney, Liver, Lung, Lymph Node, Pancreas, Skin, Spleen
IL-27	All the tissue	Blood, Brain, Eye, Heart, Intestine, Kidney, Liver, Lung, Pancreas, Skin, Spleen, Thymus

We then hypothesized that IL-35 is upregulated in inflammation. The inducibility of IL-35 has been demonstrated in human Tregs [17], and human conventional CD4^+^Foxp3^−^ T cells [28] (Table 5). However, the inducibility of IL-35 in non-T cells remains unknown. Since IL-35 nomenclature was first proposed in 2007, we analyzed the experimental reports published before 2007. We found that IL-12A and EBI3 can also be upregulated in non-T cells including immature dendritic cells, epithelial cells, smooth muscle cells, and vascular endothelial cells (Table 5). We also found that IL-35 could be upregulated in human non-T cells, such as microvascular endothelial cells, aortic smooth muscle cells, and epithelial cells by stimulations with proinflammatory cytokines tumor necrosis factor-α (TNF-α), interferon-γ (IFN-γ), and IL-1β (Table 5). In addition, in epithelial cells, TNF-α induced the upregulation of both IL-12A and EBI3 whereas type 1 T helper cell (Th1) cytokine IFN-γ only upregulated the expression of IL-12A but not EBI3. TNF-α and IFN-γ synergistically induced the upregulation of IL-12A and EBI3 expression (Table 5). These results suggest that the upregulation of IL-35 is double-gated and is controlled by two different signals, and IL-35 is only upregulated when the expressions of both subunits are induced. Moreover, in one non-IL-35 study of bacterial infection-released endotoxin lipopolysaccharide (LPS)-activated monocytes, IL-12A expression rapidly increased, peaked at 12 hours and then dropped back to background levels after 24 hours. In contrast, EBI3 showed prolonged expression kinetics, although its transcription was induced as early as 3 hours after LPS stimulation. Reaching maximal EBI3 mRNA levels around 24 hours, EBI3 was still above non-stimulated background levels after 72 hours (Table 6), suggesting that IL-12A mRNAs are less stable than that of EBI3, potentially because of mRNA degradation mechanisms. Since EBI3 can dimerize with IL-12A to form IL-35 or dimerize with IL-27 to form proinflammatory cytokine IL-27, and IL-12A can also dimerize with IL-12B to form Th1 proinflammatory cytokine IL-12, although we do not know the binding constants of these dimerizations, we found that the upregulation time of IL-12 and IL-27 were quantitatively earlier than that of IL-35 and that the scales of IL-12 and IL-27 upregulation was higher than that of IL-35. Once again, the analysis suggests that IL-35 is a responsive cytokine that is induced by inflammation. Finally, we hypothesized that if IL-35 and IL-10 are upregulated in response to proinflammatory cytokine stimulation, the expression of ubiquitously expressed TGF-β will be un-responsive to inflammation stimuli. As expected, in another non-IL-35 report, we found that proinflammatory cytokine IL-1β stimulation could upregulate IL-35 and IL-10 but not TGF-β (Table 7). Taken together, these results suggest that first, IL-35 and IL-10 are responsive anti-inflammatory cytokines whereas TGF-βs is house-keeping anti-inflammatory cytokines; second, in response to inflammation, competition of dimerization of IL-35 against dimerization of IL12 and IL27 may be one of the novel mechanisms underlying IL-35's inhibition of inflammation; and third, due to the lower expression levels, IL12A is quantitatively a limiting subunit for dimerization and upregulation of cytokine IL-35, while EBI3 is not.

**Table 5:** IL-35 subunit mRNAs have been shown to be induced by stimulation in various human cell types.

IL-35 Study	PMID	Cell Type	Treatment	Time	Induction Fold		
					IL12A	EBI3	β-Actin
Yes	20953201	Conventional CD4+Foxp3- T cells	IL-35	9 day	60	18	-
Yes	21576509	Naïve regulatory T cells	TCR/CD28 co-activation	9 day	100	150	-
No	17947455	Intestinal microvascular endothelial cells	IL-1β	8 hour	20	10	1
No	15196212	Intestinal epithelial cell line	TNF-α	8 hour	5	500	1
No	15196212	Intestinal epithelial cell line	IFN-γ	8 hour	35	1	1
No	15196212	Intestinal epithelial cell line	TNF-α+IFN-γ	8 hour	60	1000	1
No	19556516	Primary aortic smooth muscle cells	TNF-α+IFN-γ	24 hour	9	160	-
No	12446009	Immature dendritic cells	CD40L+IFN-γ	12 hour	∞	23	-

**Table 6:** IL-35 subunit mRNAs have differential induction and duration of expression in human monocytes.

IL-35 Study	PMID	Cell Type	Treatment	Time (hour)	IL12A (pg)	EBI3 (pg)	IL12B (pg)	IL27 (pg)
No	12121660	Monocyte	LPS	0	0	0	0	0
No	12121660	Monocyte	LPS	3	5	50	5000	100
No	12121660	Monocyte	LPS	6	50	90	10000	150
No	12121660	Monocyte	LPS	12	100	180	24000	300
No	12121660	Monocyte	LPS	24	10	400	2000	0
No	12121660	Monocyte	LPS	48	0	180	1000	0
No	12121660	Monocyte	LPS	72	0	140	0	0

**Table 7:** Differential response of IL-35 subunits, IL-10, and TGF-β1 in response to proinflammatory cytokine IL-1β.

IL-35 Study	PMID	Cell Type	Treatment	Time	Induction Fold				
					IL12A	EBI3	IL10	TGFB1	β-Actin
No	15130917	Umbilical vein endothelial cell	IL-1β	2.5 hour	2.2	6.1	2.3	0.9	1.0

### 3. NF-κB transcription factor has high binding frequencies in the promoter regions of IL-35 subunits IL-12A and EBI3

The upregulation of IL-35 in response to proinflammatory stimuli suggests that proinflammatory transcription factors may mediate this upregulation. Thus, we hypothesized that IL-35 subunits IL-12A and EBI3 can be regulated by specific proinflammatory transcription factors. To test this hypothesis, the promoter sequences (1,500 bases upstream of the gene transcription start site) from a control group of house-keeping genes (ACTB, GAPDH, ARHGDIA) and the promoter sequences of IL-35 subunits IL-12A, EBI3, and TGF-β1 were retrieved from the NCBI database. The promoter regions were analyzed using the transcription factor database TESS to examine the binding frequencies of 10 inflammation-regulatory transcription factor (TF) binding sites (Fig. 3B). Compared to the first control group, the promoter regions of both IL-12A and EBI3 had significantly higher frequencies of the binding sites for NF-κB (2 and 4 times respectively) (Fig. 3C). In contrast, TGF-β1 had no binding sites for NF-κB, which again supported its role as housekeeping anti-inflammatory cytokine, which correlated with the results of non-induction of TGF-β in response to NF-κB-mediated IL-1β stimulation (Table 7). Our results are consistent with previous reports on the roles of NF-κB in regulating IL-12A [29] and EBI3 [30] expression. In addition, we found that AP-1 and HSF-1 transcription factor had significantly high frequencies of binding sites in the promoter region of IL-12A whereas GR and IRF-1 had significantly high binding sites in the promoter region of EBI3. These results may shed light on the differential induction of IL-35 subunits in response to proinflammatory cytokines (Table 5), which correlates with the double-gated controls in upregulation of these IL-35 subunits.

### 4. Alternative promoter and alternative splicing regulate the structures, functions, and expressions of IL-35 and other anti-inflammatory cytokines

**Figure 4:**
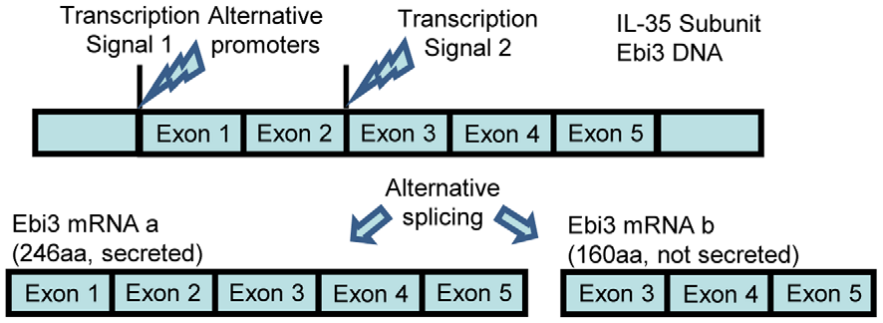
Schematic presentation of how alternative promoter and alternative splicing regulate the expression of IL-35 subunit Ebi3.

Recent findings suggest that alternatively spliced isoforms of cytokines may either enhance or antagonize the function of the other isoforms of the cytokine [31]. Since IL-35 is not constitutively expressed in most tissues, we searched for evidence that IL-35 subunit genes have alternative promoters, which serves as structural evidence for potential transcriptional induction of IL-35 in response to different stimuli [32]. We hypothesized that IL-35 and other anti-inflammatory cytokines have several alternative spliced isoforms and promoters. To test this hypothesis, we examined the AceView-NCBI database; the most comprehensive database of alternative promoters and alternatively spliced gene isoforms that is generated from experimental data of cDNA sequence analysis from tissue mRNA transcriptomes [33]. As shown in Table 8, all of the human anti-inflammatory cytokines have numerous alternatively spliced isoforms. For example, IL-35 subunit IL-12A has six isoforms, whereas IL-35 subunit EBI3 has two isoforms and two promoters (Fig. 4).

**Table 8:** Alternative promoter and alternative splicing regulate the expression and structures of anti-inflammatory cytokines.

Gene	Exons	Total Isoforms	ORF Isoforms	Secreted	Promoters
IL12A	7	6	6	4	-
EBI3	5	2	2	1	2
IL10	5	3	3	1	2
TGFB1	7	5	8	1	2
TGFB2	8	7	10	3	2
TGFB3	8	7	9	2	5

It should also be noted that one isoform of EBI3 was a secreted form, while the other was a cellular form. The potential function of intracellular isoform of EBI3 is not fully known, however in addition to the interaction with IL-12A to constitute IL-35 and its interaction with IL-27RA [34] to fulfill its cytokine function, EBI3 also interacts with four other proteins including Calnexin [35], Golgi SNAP receptor complex member 1 [34], MyoD family inhibitor [36], and SMAD family member 3 [36]. This may underlie EBI3 intracellular function. We also noticed that IL-12A has four secreted forms and two intracellular forms. Similarly, in addition to its interactions with EBI3 to constitute IL-35 and its interactions with IL-12B to constitute IL-12, IL-12A also interacts with three other proteins; Wiskott-Aldrich syndrome protein (WASP)-family member 1 [37], CD28 [38], and IL-8 [37], which may provide insight for IL-12A intracellular and/or cytokine functions. As we and others reported previously, a similar multi-functional cytokine can be seen in translationally controlled tumor protein (TCTP), which has an intracellular function of anti-apoptosis [39] and a secreted cytokine function of histamine release [40].

Of note, IL-35 subunit IL-12A, TGF-β2, and TGF-β3 have more than one secreted isoforms with isoform-specific protein sequences. Potential binding differences of these secreted cytokine isoforms to their receptors could lead to different functions. In addition, all of anti-inflammatory cytokines have alternative promoters, suggesting the importance of tissue-specific and/or stimulation-specific transcriptional mechanisms in mediating the transcriptional upregulation of the genes via alternative promoters [32]. Of note, there was no alternative promoter data on IL-12A in the database. Future work is needed to map out the detailed sequence requirements in the alternative promoters in response to various stimuli.

### 5. Higher expression of IL-35 could be induced by higher hypomethylation status in tissues

**Figure 5:**
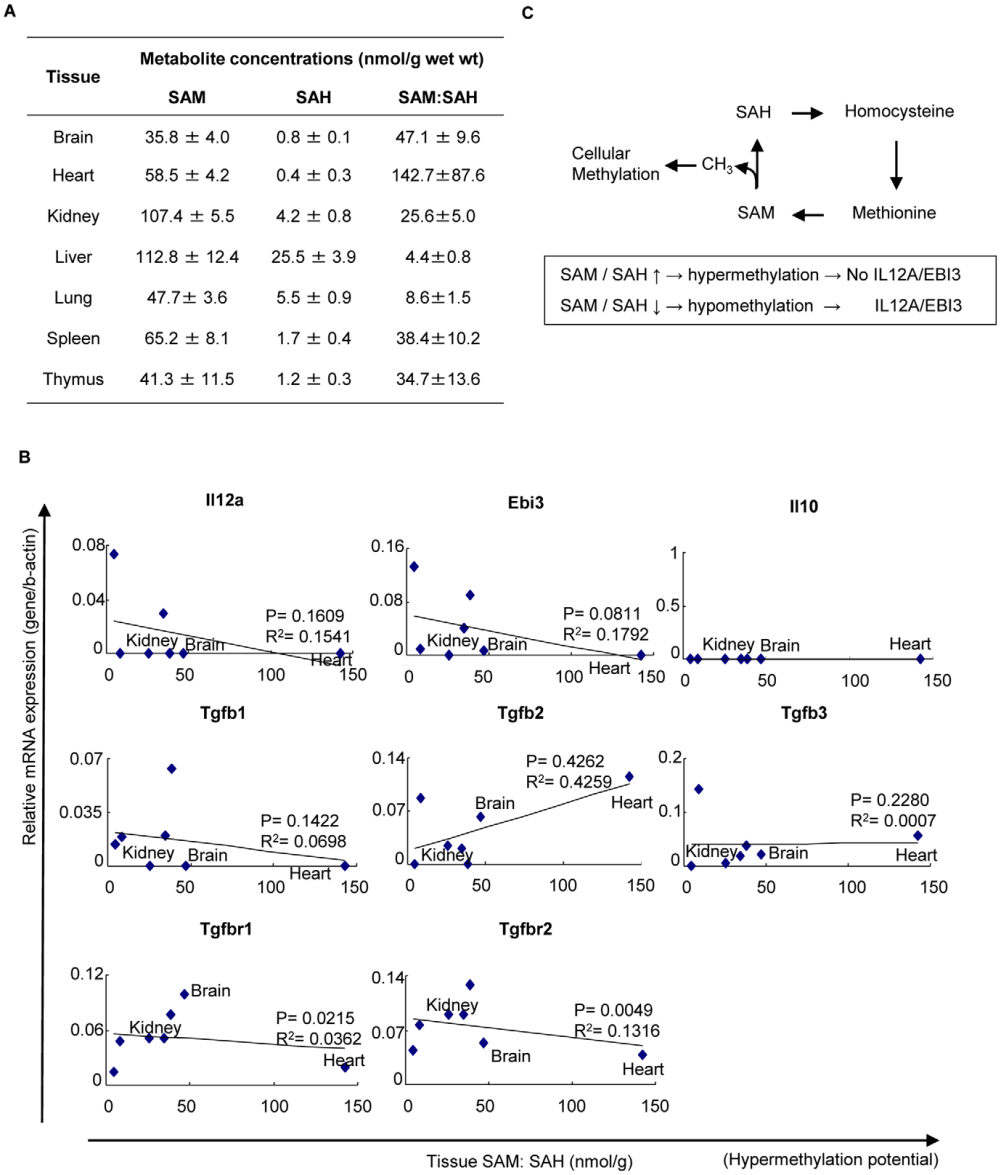
Higher hypomethylation status is positively associated with higher expression of IL-35 gene. A. Concentrations of SAM and SAH in mouse tissues were previously examined by Ueland et al. B. Correlation of suppressive cytokines and TGF-β receptors with SAM/SAH ratios in mouse tissues. C. Schematic presentation of how IL-35 may be regulated by methylation status. S-Adenosylhomocysteine (SAH) and S-Adenosylmethionine (SAM) are intermediate metabolites of homocysteine-methionine metabolism cycle. SAH is a potent inhibitor of cellular methylation. High SAM/SAH ratio is associated with hypermethylation of DNA and no IL12A/Ebi3 expression. Low SAM/SAH ratio is associated with hypomethylation of DNA and Ebi3 can be expressed.

Previous reports showed that epigenetic mechanisms, including methylation and demethylation, control T helper cell differentiation and cytokine generation [41]. As we discussed in our recent review [42], the ratio of cellular methylation donor S-adenosylmethionine (SAM) levels over S-adenosylhomocysteine (SAH) levels is an important metabolic indicator of cellular methylation status [43], [44]. A higher SAM/SAH ratio suggests a higher methylation status than normal (hypermethylation) whereas a lower SAM/SAH ratio indicates a lower methylation status than normal (hypomethylation). A previous report showed that feeding rats with SAM, a methyl donor, inhibits the expression of TGF-βR1 and TGF-βR2 [45], suggesting that intracellular global methylation status regulates anti-inflammatory cytokine signaling. We hypothesized that intracellular methylation/demethylation status, a major metabolic stress-related epigenetic modification [46], may regulate the expression of IL-35 and other anti-inflammatory cytokines and the associated receptors in tissues. To test this hypothesis, we used tissue concentrations of SAH and SAM and the ratio of SAM over SAH in seven mouse tissues; brain, heart, kidney, liver, lung, and thymus [20], which were reported previously by Ueland's group [22] (Fig. 5A). We performed multivariable regression analyses to determine the effect of cellular methylation, indicated by the SAM/SAH ratio, on the expression of anti-inflammatory cytokines and receptors. As shown in Figure 5B, the SAM/SAH ratios negatively correlated with the expression levels of TGF-βR1 and TGF-βR2 (*p*<0.05). This result corresponds with previous experimental studies [45], which suggest that our approach was feasible in examining the role of hypomethylation in regulating the expression of anti-inflammatory cytokines, and that DNA methylation status in cells can determine the functional status of TGF-β signaling [47]. Notably, the tissue expression of the two subunits of IL-35, IL-12a, and EBI3, were increased as the tissue SAM/SAH ratios were decreased (*p* = 0.16 or 0.06). Although these P values were >0.05, these results suggest that hypomethylation, induced by metabolic stress including pro-atherogenic risk factor hyperhomocysteinemia [20], could induce the expression of IL-35 in mice (Fig. 5C). In support of our regression results, our analysis on previously reported microarray experimental data in a non-IL-35 related study showed that treatment of human cells with azacytidine (AZC), a DNA methyl transferase inhibitor and a hypomethylation inducer, induces the upregulation of IL-35 subunit transcripts [48]. Similarly, since global DNA hypomethylation has been observed in lupus, an autoimmune inflammatory disease [49], taken together, our results suggest that DNA hypomethylation-induced expression of IL-35 may result from a negative feedback mechanism during full-blown inflammation.

### 6. The expressions of IL-35 can be regulated by an AU-rich element-mediated mRNA degradation mechanism

**Figure 6:**
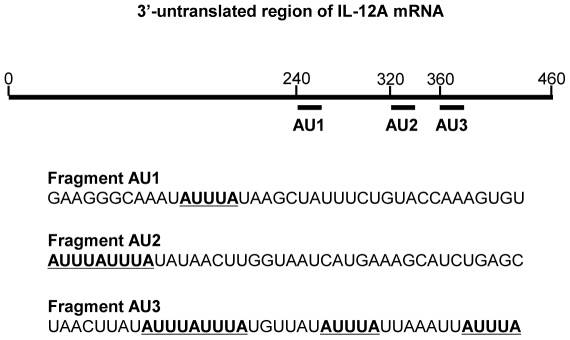
Positions of AUUA sequences in 3′-UTR of IL-12A mRNA are indicated.

Although IL-35 subunits can be induced by proinflammatory cytokine in many cell types, their mRNAs have differential expression kinetics within the same cell type under identical conditions (Table 6). We argued that non-constitutive expression status of IL-35 in tissues may be realized by quick degradation following their upregulation presumably via mRNA degradation mechanisms. We hypothesized that RNA binding proteins may regulate the mRNA stability of IL-35 subunits. Thus, we searched for evidence for whether IL-35 subunit mRNA 3′ untranslated region (3′UTR) had specific structural features mediating quick degradation. Using a web-based AU-rich element mRNA database [50], we analyzed all the mRNA 3′untranslated regions (UTRs) of anti-inflammatory cytokines in the UTR database UTRdb (http:// utrdb.ba.itb.cnr.it/search). As shown in Table 9, IL-12A and IL-10 contained AU-rich elements in their mRNA 3′UTRs, suggesting that IL-35 (also IL-12) and IL-10 are under regulation by the AU-rich element-mediated mRNA degradation mechanism. In contrast, no AU-rich elements were found in 3′UTRs of TGF-βs. Since our data showed that IL-12A is a limiting factor for upregulation of IL-35 (Table 6), potential quick degradation of IL-35 subunit IL-12A via an AU-rich mechanism correlated with the non-constitutive expression status of IL-35 (Fig. 6). These suggest that AU-rich element-mediated mRNA degradation may participate in regulating mRNA stability of IL-35. Our results were supported by a previous experimental report on the role of Tristetraprolin, an AU-rich element binding protein, in regulating IL-12A (IL-35 subunit) expression [51].

**Table 9:** 3′-untranslated region in mRNA of anti-inflammatory cytokines in human contain signals for RNA protein binding.

Human Gene	Length bp	Signal	Class	Cluster
IL12A	460	ARE	II	3
EBI3	579	-	-	-
IL10	1033	ARE	I	5
TGFB1	728	-	-	-
TGFB2	6325	-	-	-
TGBF3	2150	-	-	-

### 7. The microRNAs that target IL-12A and EBI3 mRNA 3′UTRs are not shared, suggesting that the expressions of these two subunits of IL-35 may be regulated by different microRNAs

**Figure 7:**
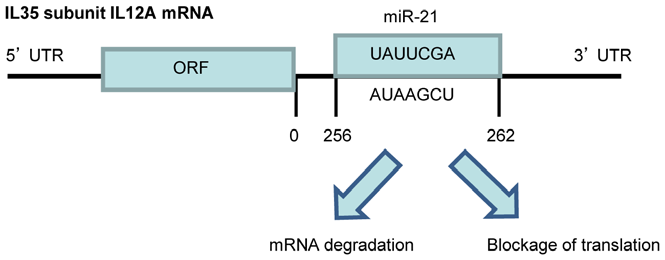
Schematic presentation of how miR-21 may regulate the mRNA stability of IL-35 subunit IL12A.

MicroRNAs (MiRNAs or MiRs) are a newly characterized class of short (18–24 nucleotide long) [52], endogenous, and non-coding RNAs, which contribute to the development of particular disease states through the regulation of diverse biological processes such as cell growth, differentiation, proliferation, and apoptosis [53]. This regulation occurs through base-pairing with messenger RNAs (mRNAs) predominately at the 3′UTR [54], [55], and leads to degradation or inhibition of mRNA translation [56]. A sequence analysis identified miR-16 as possessing complementary sequence to the canonical AUUUA, which demonstrates a potential role for this microRNA to modulate the stability of mRNAs with this AU-rich element [57]. Since we found this AU-rich element in the 3′UTR of IL-35 subunit IL-12A and IL-10, we hypothesized that mRNAs of IL-35 and other anti-inflammatory cytokines contain the structures in their 3′UTRs for microRNA binding and regulation. To examine this hypothesis, we used the online microRNA target prediction software, TargetScan (http://www.targetscan.org/) developed in the Massachusetts Institutes of Technology. To ensure that the predicted microRNAs have the binding quality equivalent to that of experimentally verified microRNAs, we reasoned that there are certain shared binding features between predicted microRNAs and targeted 3′UTRs of mRNAs that can be reflected in the context value and context percentage. To test this hypothesis, the confidence intervals for context value (the mean ±2×SD = −0.25±0.12) and for context percentage (76.07±19.07) were generated, respectively, from 45 interactions between 27 experimentally verified human microRNAs and 36 different genes (not shown) within Tarbase, an online database of experimentally verified microRNAs (http://diana.cslab.ece.ntua.gr/tarbase/) [58]. These human microRNAs were all confirmed using luciferase reporter assays and had been found to effectively target a single unique mRNA sequence. As shown in Table 10, microRNAs may regulate anti-inflammatory cytokines. We found that 3 microRNAs on the list have more than one binding site either within cognate target 3′UTR or within other genes. This finding correlated with others' report that some microRNAs have numerous mRNA targets [59]. Of note, the microRNAs targeting IL-35 subunits IL-12A and EBI3 mRNA 3′UTRs were not shared, suggesting once again that the expressions of these two subunits of IL-35 are differentially regulated by different microRNAs in a “double-gated” manner. Furthermore, 2 predicted microRNA target including IL12A on our list have been confirmed in experiments (Table 11). These results suggest that microRNAs may inhibit the mRNA stability and translation of anti-inflammatory cytokines including IL-35 (Fig. 7), either independently or via interactions with RNA binding protein in various tissues.

**Table 10:** Predicated microRNAs that could bind to anti-inflammatory cytokines.

mRNA	MicroRNA	Position	mRNA	MicroRNA	Position
IL12A	miR-21	256–262	IL10	-	-
	miR-590-5p	256–262	TGFB1	miR-139-5p	292–298
EBI3	miR-576-5p	277–283	TGFB2	miR-141	109–115
	miR-136	186–192		miR-200 A	109–115
	miR-185	202–208	TGFB3	miR-631	772–778
	miR-548m	211–217		miR-875-5p	960–966
	miR-548m	217–223		miR-511	967–974
	miR-128	251–257		miR-520d-5p	997–1003
	miR-27b	252–258		miR-524-5p	997–1003
	miR-27a	252–258		miR-876-3p	32–38
	miR-552	317–323		miR-1291	365–371
	miR-663b	388–394		miR-1291	452–458
	miR-644	390–396		miR-649	486–492
	miR-1303	409–415		miR-644	926–932

**Table 11:** Some predicted MicroRNA targets listed above have been confirmed in experiments.

MicroRNA	Reported Target	Reported Function	PMID
miR-21	IL35/IL12A	Introduction of pre-miR-21 dose dependently inhibited cellular expression of a reporter vector harboring the 3′-untranslated region of IL-12p35.	19342679
miR-200A	TGFB2	miR-200a downregulated the expression of TGF-β2 via direct interaction with the its 3′ untranslated region	20952520

## Discussion

Since 2007, a new anti-inflammatory/immunosuppressive cytokine, IL-35, has been defined [16], [60], [61]. Formed by heterodimerization of Epstein-Barr virus-induced gene 3 (EBI3) protein with the IL-12 p35 subunit (IL-12A) [15], IL-35 inhibits inflammation in various autoimmunity models such as experimental colitis [16], [62], collagen-induced autoimmune arthritis [61], autoimmune demyelination in central nervous system [63], and type 2 T helper cell (Th2)-mediated allergic asthma [64]. Although secretion of IL-35 has only been confirmed in Tregs [15], the contributions of IL-35 generated from tissues and cells other than T cells under non-stimulated conditions remains poorly identified. In an effort to bridge this knowledge gap we used a new experimental database mining and statistical analysis technique to determine the tissue expression of anti-inflammatory cytokines and receptors including novel cytokine IL-35 and made the following findings: *1)* anti-inflammatory cytokines IL-35 and IL-10 are not constitutively expressed in most tissues whereas TGF-βs have higher expression levels in non-stimulated tissues; *2)* IL-35 receptor subunit IL-6ST, IL-10RA, IL-10RB, TGF-βR1, and TGF-βR2 receptor complex are constitutively expressed in cardiovascular and other tissues; *3)* NF-κB transcription factor has higher binding frequencies in the promoter region of IL-35 subunits; *4)* alternative promoter and alternative splicing regulate the structures and expressions of IL-35 and other anti-inflammatory cytokines and receptors; *5)* higher expression of IL-35 could be induced with higher hypomethylation status. The higher binding frequencies of NF-κB-transcription factor in IL-35 promoters, alternative promoters, along with higher expression of IL-35 induced by hypomethylation all suggest that the expression of IL-35 is inducible via several mechanisms. To support our results, we also presented the reported experimental evidence that IL-35 is indeed induced by stimulations of various proinflammatory cytokines and bacterial endotoxin LPS in vascular endothelial cells, smooth muscle cells and monocytes; *6)* the expressions of IL-35 and IL-10 could be regulated by AU-rich element-mediated mRNA degradation mechanism; and *7)* the two subunits of IL-35 are subjected to regulation by different microRNAs. The last two structural evidences indicate that IL-35 can be degraded quickly by 3′UTR-mediated mechanisms, which correlates with the non-constitutive expression of IL-35 in tissues.

It is worth pointing out that the data retrieved from the expression sequence tag (EST) database analyzed in this study is more precise than that detected with traditional approaches including Northern blot analysis and PCR analysis due to the un-biased cDNA cloning and DNA sequencing procedures of EST database deposits [21]. Thus, the expression patterns of IL-35, other anti-inflammatory cytokines, and receptors are experimentally-based and precise.

Transcription factors (TFs) are master genes which control the expression of other genes. It is well-accepted that multiple binding sites for a given TF within a promoter will increase the likelihood of actual binding [65]. The most physiologically relevant TFs will bind to the putative core promoter region (1,500 base pairs upstream of the transcription start site) to fulfill their functions [66]. Our strategy to identify TF binding profiles and transcriptional signaling is an important advance in merging bioinformatics and experimental science. This study, together with our previous database mining work [20], [21], [23], utilized novel database mining techniques to identify disease-related signaling pathways. Our research method is featured as; *(1)* hypothesis-driven, *(2)* intensively grounded in the literature, *(3)* panoramic and integrative for gene and TF regulation, *(4)* based on the NCBI experimental databases, *(5)* inclusive of well-characterized TFs in the searchable database TESS; *(6)* statistically rigorous analysis of available public databases, and *(7)* experimentally verified.

Alternative promoters play an important role in gene transcription in response to tissue/cell-specific and stimulation-specific transcription signaling [67]. One of the best examples of multiple promoter usage is fibroblast growth factor-1 (FGF1) transcription, which is controlled by at least four distinct promoters in a tissue-specific manner. The 1.A and 1.B promoters of FGF1 are constitutively active in their respective cell types. In contrast, different biological response modifiers, including serum and transforming growth factor-β, can induce the 1.C and 1.D promoters of FGF1 [67]. Of note, our results showed that most anti-inflammatory cytokines and cytokine receptors have more than one promoter, suggesting the capacity of these genes to respond to tissue-/cell-specific and stimulation-specific transcription signaling [67]. Although endothelial cells and vascular smooth muscle cells also generate various inflammation-regulating cytokines [19], the lack of a cell type-specific gene expression database prevents the analysis of databases in cell-specific manner. When the detailed sequences become available, it will be possible to compare the transcription factor binding profiles in the alternative promoters of the same genes.

**Figure 8:**
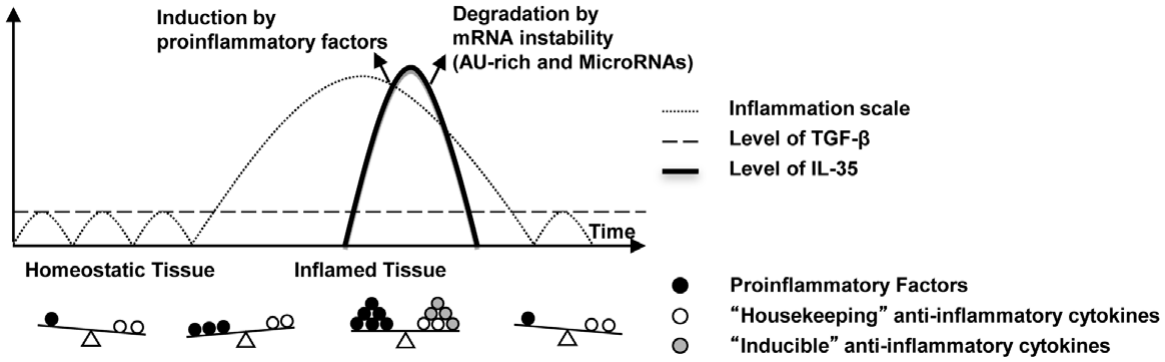
A new working model of responsive anti-inflammatory cytokine and housekeeping cytokine. Homeostatic tissues express “house-keeping” anti-inflammatory cytokines TGF-β1, TGF-β2, TGF-β3 to prevent it from initiation of inflammation. When tissues get inflamed, proinflammatory factors may stimulate tissues to express “responsive” anti-inflammatory cytokines such as IL-35 by specific transcription factors to counteract inflammation response. Furthermore, ARE binding proteins and MicroRNAs are responsible of the quick degradation of IL-35 mRNA afterwards, by which IL-35 achieve non-constitutive expression status in tissues again.

To summarize our results, we propose a new system of categorizing anti-inflammatory cytokines (Fig. 8) based on the following three criteria including *(1)* constitutive or non-constitutive expression in tissues, *(2)* non-responsiveness or responsiveness to proinflammatory stimuli, and *(3)* acceleration or no acceleration of autoimmune and inflammation by cytokine gene deficiency. We categorize anti-inflammatory cytokines into two groups: first, the house-keeping cytokines are defined with constitutive expression in tissues, non-responsiveness to proinflammatory stimuli, and acceleration of autoimmune and inflammation in the absence of the genes, such as TGF-βs; second, the definitions of responsive cytokines include cytokines with non-constitutive expression in tissues, responsiveness to proinflammatory stimuli and no acceleration of autoimmune and inflammation in the absence of the genes, including IL-35. IL-35 is not constitutively expressed in most tissues similar to IL-10. Instead it is upregulated, by hypomethylation and other proinflammatory signals, in human tissues and most mouse tissues except mouse blood, bone marrow, liver, and thymus. In addition, the expression and structure of IL-35 are under various regulations including NF-κB transcription factors, alternative promoter, alternative splicing, and mRNA degradation via AU-rich-dependent and microRNA-dependent mechanisms. These mechanisms underlie the upregulation and quick degradation of IL-35. Our new working model and new system in categorizing anti-inflammatory cytokines provide important insight into the following two important issues: first, how anti-inflammatory cytokines share their duties: the house-keeping cytokines, such as TGF-βs, inhibit the initiation of inflammation whereas the responsive cytokines including novel cytokine IL-35 suppress full-blown inflammation; and second how these two groups of anti-inflammatory cytokines orchestrate their roles in suppressing inflammation in different stages in various tissues and systems. Our findings are significant for future design of novel anti-inflammatory/immunosuppressive therapies.

## Supporting Information

Figure S1 **The gene expression profiles of anti-inflammatory cytokine receptors in human and mouse tissues.**
(PPT)

Table S1 **The Unigene ID of human and mouse genes that were examined.**
(PPT)
