# Supplementary material for: IL-35 Is a Novel Responsive Anti-inflammatory Cytokine — A New System of Categorizing Anti-inflammatory Cytokines
Source: PLoS One. 2012 Mar 16;7(3):e33628. doi: 10.1371/journal.pone.0033628 (PMC3306427; doi:10.1371/journal.pone.0033628)
Supplement: Figure S1 — The gene expression profiles of anti-inflammatory cytokine receptors in human and mouse tissues. (PPT) [file pone.0033628.s001.ppt]

## Slide 1
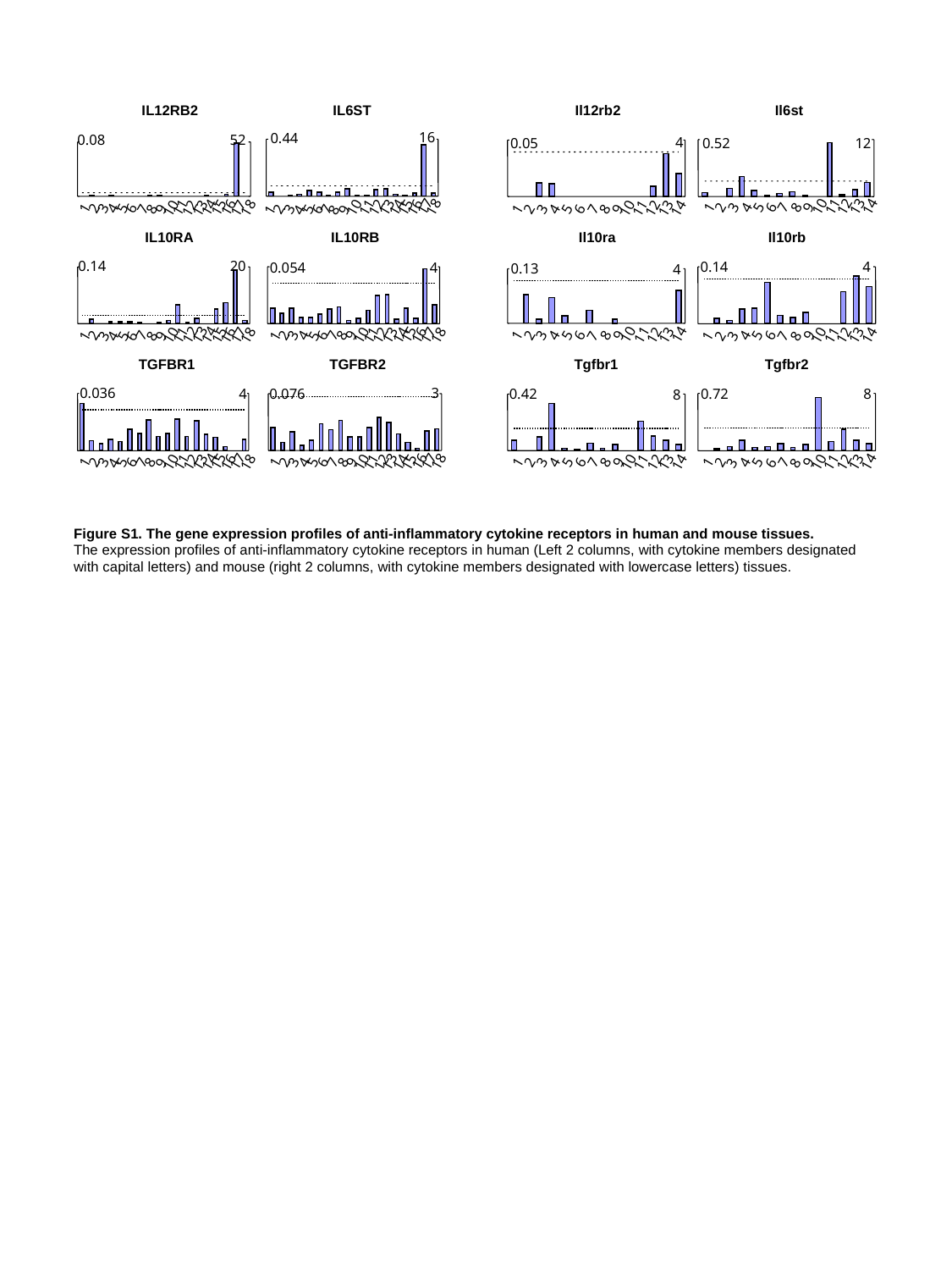

IL6ST
16
0.44
17
12
15
18
14
11
16
10
13
7
2
5
3
6
1
4
8
9
IL12RB2
Il12rb2
4
0.05
10
13
14
11
12
4
5
6
1
2
3
7
8
9
Il6st
0.52
12
10
11
12
13
14
1
2
3
4
6
7
9
5
8
0.08
52
11
14
15
16
10
12
17
18
13
3
6
1
4
5
2
8
9
7
IL10RA
0.14
20
12
13
14
10
11
15
16
17
18
2
7
1
3
4
6
8
9
5
IL10RB
0.054
4
15
11
12
16
10
13
17
18
14
2
3
4
9
6
7
8
1
5
Il10ra
0.13
4
10
14
11
12
13
6
1
2
3
4
5
8
9
7
Il10rb
0.14
4
11
12
13
10
14
4
5
6
9
1
2
3
7
8
TGFBR1
0.036
4
15
17
10
13
14
16
11
12
18
5
8
1
2
3
6
7
4
9
TGFBR2
3
0.076
16
17
18
10
13
15
11
12
14
1
2
3
4
5
6
7
9
8
Tgfbr1
0.42
8
10
11
12
13
14
1
2
3
4
5
6
7
8
9
Tgfbr2
0.72
8
12
13
14
10
11
1
2
4
5
7
9
3
6
8
Figure S1. The gene expression profiles of anti-inflammatory cytokine receptors in human and mouse tissues.
The expression profiles of anti-inflammatory cytokine receptors in human (Left 2 columns, with cytokine members designated with capital letters) and mouse (right 2 columns, with cytokine members designated with lowercase letters) tissues.
