# Supplementary material for: IL-35 Is a Novel Responsive Anti-inflammatory Cytokine — A New System of Categorizing Anti-inflammatory Cytokines
Source: PLoS One. 2012 Mar 16;7(3):e33628. doi: 10.1371/journal.pone.0033628 (PMC3306427; doi:10.1371/journal.pone.0033628)
Supplement: Table S1 — The Unigene ID of human and mouse genes that were examined. (PPT) [file pone.0033628.s002.ppt]

## Slide 1
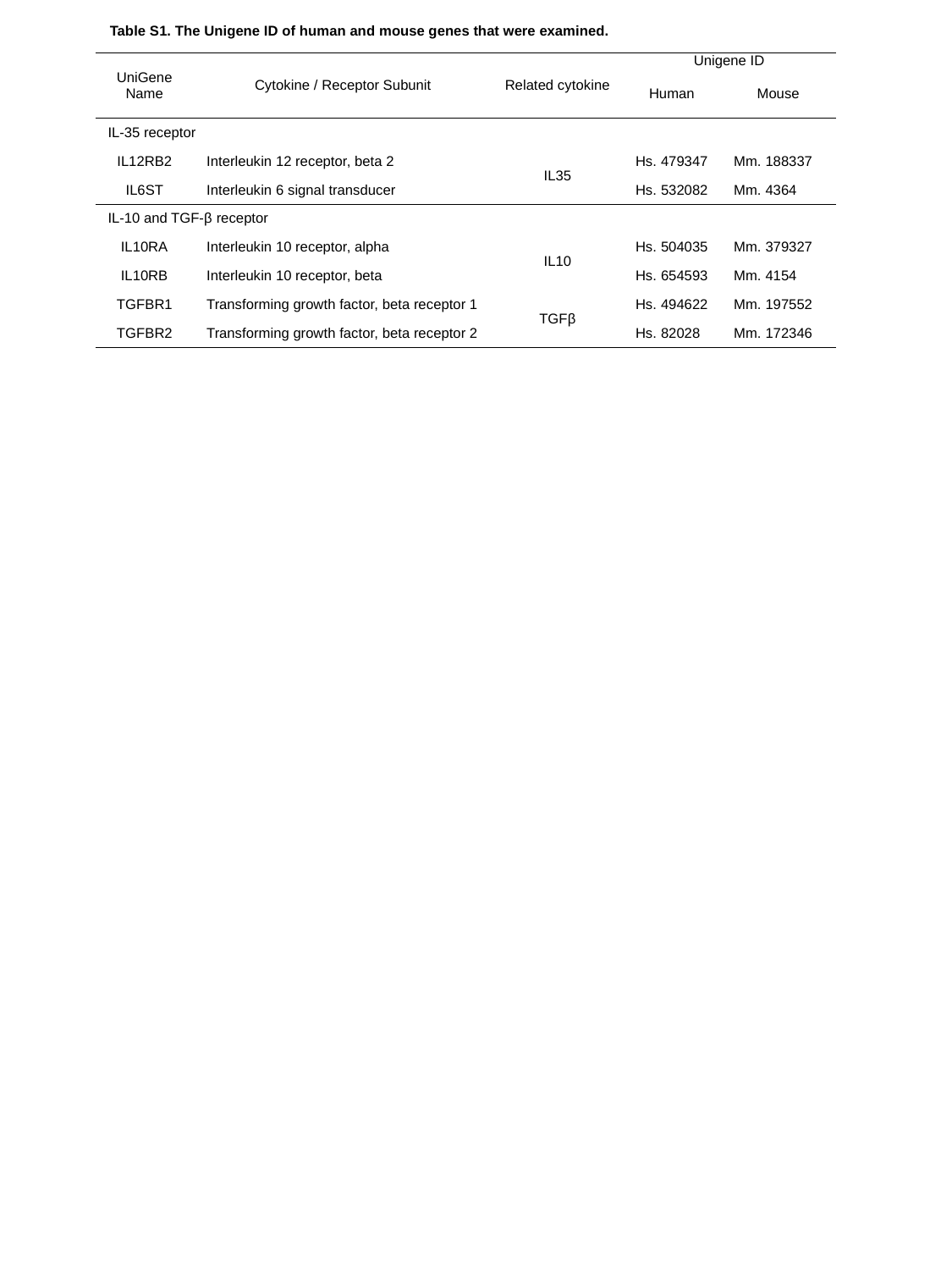

Table S1. The Unigene ID of human and mouse genes that were examined.
| UniGene Name | Cytokine / Receptor Subunit | Related cytokine | Unigene ID | |
| --- | --- | --- | --- | --- |
| | | | Human | Mouse |
| IL-35 receptor | | | | |
| IL12RB2 | Interleukin 12 receptor, beta 2 | IL35 | Hs. 479347 | Mm. 188337 |
| IL6ST | Interleukin 6 signal transducer | | Hs. 532082 | Mm. 4364 |
| IL-10 and TGF-β receptor | | | | |
| IL10RA | Interleukin 10 receptor, alpha | IL10 | Hs. 504035 | Mm. 379327 |
| IL10RB | Interleukin 10 receptor, beta | | Hs. 654593 | Mm. 4154 |
| TGFBR1 | Transforming growth factor, beta receptor 1 | TGFβ | Hs. 494622 | Mm. 197552 |
| TGFBR2 | Transforming growth factor, beta receptor 2 | | Hs. 82028 | Mm. 172346 |
